# Bone age assessment based on deep neural networks with annotation-free cascaded critical bone region extraction

**DOI:** 10.3389/frai.2023.1142895

**Published:** 2023-03-02

**Authors:** Zhangyong Li, Wang Chen, Yang Ju, Yong Chen, Zhengjun Hou, Xinwei Li, Yuhao Jiang

**Affiliations:** ^1^Chongqing Engineering Research Center of Medical Electronics and Information Technology, Chongqing University of Posts and Telecommunications, Chongqing, China; ^2^Department of Mechanical Science and Engineering, Graduate School of Engineering, Nagoya University, Nagoya, Japan

**Keywords:** bone age assessment, two-stage deep learning method, critical bone region extraction network, gender-assisted bone age estimation network, visual heat map

## Abstract

Bone age assessment (BAA) from hand radiographs is crucial for diagnosing endocrinology disorders in adolescents and supplying therapeutic investigation. In practice, due to the conventional clinical assessment being a subjective estimation, the accuracy of BAA relies highly on the pediatrician's professionalism and experience. Recently, many deep learning methods have been proposed for the automatic estimation of bone age and had good results. However, these methods do not exploit sufficient discriminative information or require additional manual annotations of critical bone regions that are important biological identifiers in skeletal maturity, which may restrict the clinical application of these approaches. In this research, we propose a novel two-stage deep learning method for BAA without any manual region annotation, which consists of a cascaded critical bone region extraction network and a gender-assisted bone age estimation network. First, the cascaded critical bone region extraction network automatically and sequentially locates two discriminative bone regions *via* the visual heat maps. Second, in order to obtain an accurate BAA, the extracted critical bone regions are fed into the gender-assisted bone age estimation network. The results showed that the proposed method achieved a mean absolute error (MAE) of 5.45 months on the public dataset Radiological Society of North America (RSNA) and 3.34 months on our private dataset.

## Introduction

**Figure 1:**
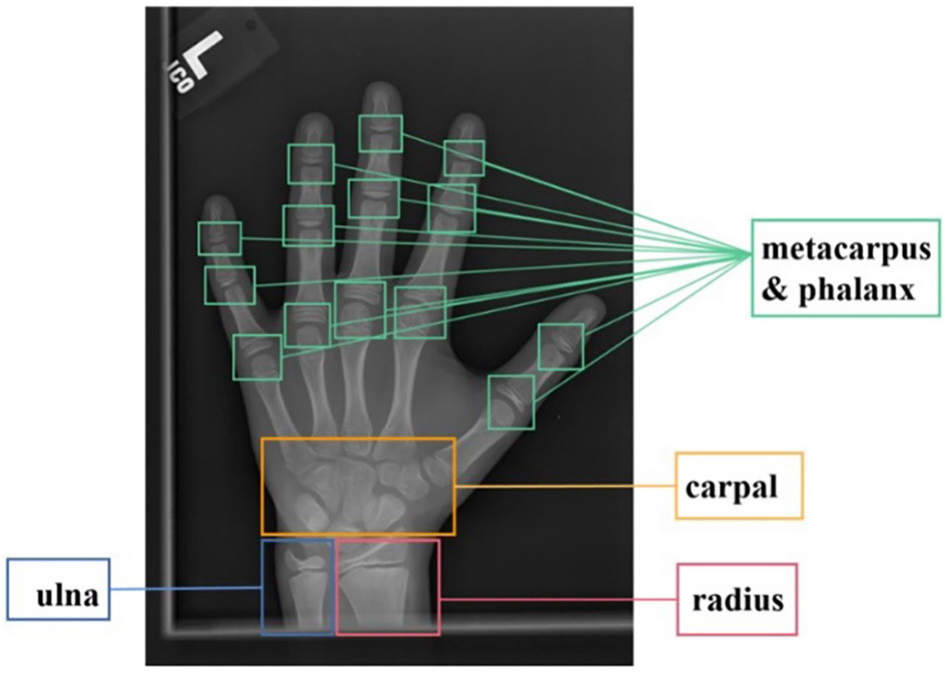
ROIs of bone X-ray images for bone age assessment.

Bone age assessment refers to a clinical application that is widely used in pediatric radiology, therapeutic estimation of endocrinology disorders, and judgment of children's growth (Poznanski et al., 1978; Carty and Journal, 2002; Thodberg et al., 2009). The BAA technique usually estimates the skeletal maturity of bones based on left-hand radiographs, for the reason that the bone ossification levels of the non-dominant hand reveal the bone maturity. The conventional standard bone age assessment methods are Greulich-Pyle (G&P) method (Greulich and Pyle, 1959) and Tanner Whitehouse (TW) method (Morris, 1976). The G&P method compares the radiographs with the reference atlas until the most similar atlas is selected, and the labeled age of the selected atlas represents the estimated bone age. Compared to the G&P method, the TW method is more complex, it analyzes specific regions of interest (ROIs, which consist of the radius, ulna, carpal, and metacarpus & phalanx bones, as shown in Figure 1), and bone age is evaluated individually through a scoring mechanism, rather than based on the entire radiograph. Due to the critical anatomical features in ROIs playing important roles in BAA, each ROI is assessed by a numerical scoring system, and the final bone age is estimated by averaging all ROI scores. Nonetheless, these conventional evaluating works are generally conducted by experienced radiologists or pediatricians through visual inspection and manual annotation, which is not only tedious, heavy workload, and long time-consuming, but also faces the influence of different doctors with different standards. As a result, automated assessment approaches for BAA are currently receiving increasing attention.

In recent years, many reported studies have shown that deep learning-based models and architectures were good at medical image segmentation, classification, and prediction (Anthimopoulos et al., 2016; Duan et al., 2018; Sanchez-Riera et al., 2018; Tajbakhsh et al., 2020). The existing deep learning models, such as convolutional neural networks (CNN), deep belief networks (DBN), and recurrent neural networks (RNN), demonstrate effective performance for image recognition, segmentation, prediction, and classification.

Meanwhile, many deep learning approaches have become increasingly popular and have been reported for automatic bone age estimation (Halabi et al., 2018), on account that they can extract the ossification patterns from the bone images and complete the complicated bone age approximation. Automatically deep learning methods for BAA could overcome the issues that refer to as time-consuming, inherent subjectivity of human interpretation, inter-operator, and intra-operator variations (Lee and Lee, 2021). Liu et al. (2019) applied learning-to-rank methods to evaluation problems and proposed a new architecture VGG-U-Net. Chu et al. (2018) proposed a fully convolutional network, U-Net-VGG, that can quickly obtain accurate masks for all input images and transform the ordered regression problem of bone age assessment into a K-1 binary classification subproblem to reduce the effect of outliers. Among all the deep learning models, the CNN model is most usually employed to segment and classify bone age, for the reason that CNN model could extract features and utilizes global or local information of carpal bones, which improves the robustness of bone age assessment and reduces the mean error of the assessment results. Nevertheless, accurate automatic bone age assessment is still a challenging task.

Due to the ROIs (metacarpus & phalanx, carpal, ulna, and radius bones) playing important roles in bone age estimation, the previous deep learning approaches for BAA could be separated into two classes, including annotation-free methods and annotation-based methods. The former annotation-free methods employ the whole hand radiographs as input and usually fed them into the end-to-end and single-stage model for estimated bone age directly. Spampinato et al. (2017) built Bonet with an extra deformation layer to get low and middle-level pattern maps, and it achieves the MAE of 9.6 months on the RSNA dataset. Larson et al. (2017) built a deep residual network structure (ResNet50) for automated bone age recognition based on the G&P mapping method, and it achieves the MAE of 6.24 months on the RSNA dataset. This network quantitatively evaluates the bone age, and it also qualitatively identifies the most sensitive regions of each image. Pan et al. (2020) also used a U-Net model to segment hand mask images from raw X-ray images, and it is a deep active learning technique that could reduce the annotation burden, and it achieves the MAE of 8.59 months on the RSNA dataset. The method proposed a new active learning framework for hand radiograph segmentation *via* a few labeled datasets. As a result, these above annotation-free methods demonstrate poor bone age estimation and are explainable.

The latter annotation-based methods use the processed images with manual annotations of bounding boxes as input. These strategies could extract features in specific regions according to prior knowledge and then produce the estimated age. Iglovikov et al. (2017) employed a U-net structure to extract important point areas based on manually labeled hand masks and achieved a performance of 6.3 months MAE for men and 6.49 months MAE for women. Escobar et al. (2019) utilized the input hand images that annotated key points with extra manual labeled boundary boxes into the training network, as a result, they obtained an effective performance in RSNA with 4.14 months MAE. Ren et al. (2018) utilized Faster-RCNN to figure out the hand foreground and used Inception-V3 architecture for age recognition, which achieved a result with 5.2 months MAE on the RSNA dataset. Son et al. (2019) localized the areas within 13 different bones based on the TW3 method and evaluated the bone age, which achieved a result with 5.52 months MAE on the RSNA dataset.

The annotation-based methods with the additional manual annotations generally show better performance and higher accuracy than the annotation-free methods. The methods that use the original image as input do not fully exploit the discriminative local information and ignore the fine-grained analysis of specific regions, so the accuracy and interpretability of these methods tend to be poor. Manual annotation is also time-consuming and has hindered the translation of experimental methods into clinical applications.

Given the problems existing in existing research methods and the shortcomings of the most commonly used G&P and TW methods, our two-stage bone age evaluation method of positioning + prediction not only considers the weight of different bone areas but also does not require physicians to spend a lot of time on bone age analysis as the TW method does, which also reduces the impact of subjective differences among different evaluators. At the same time, we used Grad-CAM to visually present the imaging content of the bone region in the positioning stage and compared the extracted hand bone region with the observation area of the traditional atlas method, which has better clinical acceptance for physicians.

We have proposed a two-step method for fully automatic bone age estimation based on X-ray images of hand bones without any annotation. The architecture consists of a key bone region localization network and a bone age estimation network. In the first step, an enhanced Inception V3 network with the Convolutional Block Attention Module (CBAM) is implemented to automatically identify and localize the critical regions of hand bone images *via* visualized heat maps. In the second step, the Xception and ResNet50 networks were used to extract the bone region features of the carpal and metacarpophalangeal regions, respectively. The modal fusion information was then obtained by dynamically exchanging feature mappings. We feed the gender information into the age estimation network as an additional input, which helps to optimize the deep learning network and improve the estimation performance.

The contributions of this paper are summarized as follows: (1) This method proposes an effective strategy to identify the critical bone regions for bone age estimation, which could encourage the model to focus on the features of critical regions and weaken the influence of irrelevant regions. (2) The proposed method feeds the gender information into the age assessment network as additional input, which improves the generalization of the bone age assessment network. (3) This two-stage structure, including critical bone region localization and bone age estimation, is more interpretable and increases the clinical acceptance of the proposed method.

## Materials and methods

### Dataset

The RSNA dataset is public and collected by the Radiological Society of North America (RSNA), it consists of 14,236 hand bone radiographs, including 12,611 training datasets, 1,425 validation datasets, and 200 test datasets (Halabi et al., 2018). Among the 12,611 training datasets, there are 5,778 female subjects and 6,833 male subjects, with an age range of 1–228 months, mainly specific to children aged 5–15 years, and each image is labeled with the true bone age. The experimental data can be downloaded on the website https://www.kaggle.com/kmader/rsna-bone-age.

The CQJTJ dataset is an additional dataset collected by the radiology department of Chongqing Jintongjia Children's Hospital. It consists of 3,551 left-hand X-ray images and corresponding clinical reports. Among these images, some are collected from the same subjects at different periods, 1,502 images are obtained from male subjects and 1,949 images are obtained from female subjects, with an age range of 13–218 months, mainly for children aged 4–16 years.

### Network architecture for bone age assessment

**Figure 2:**
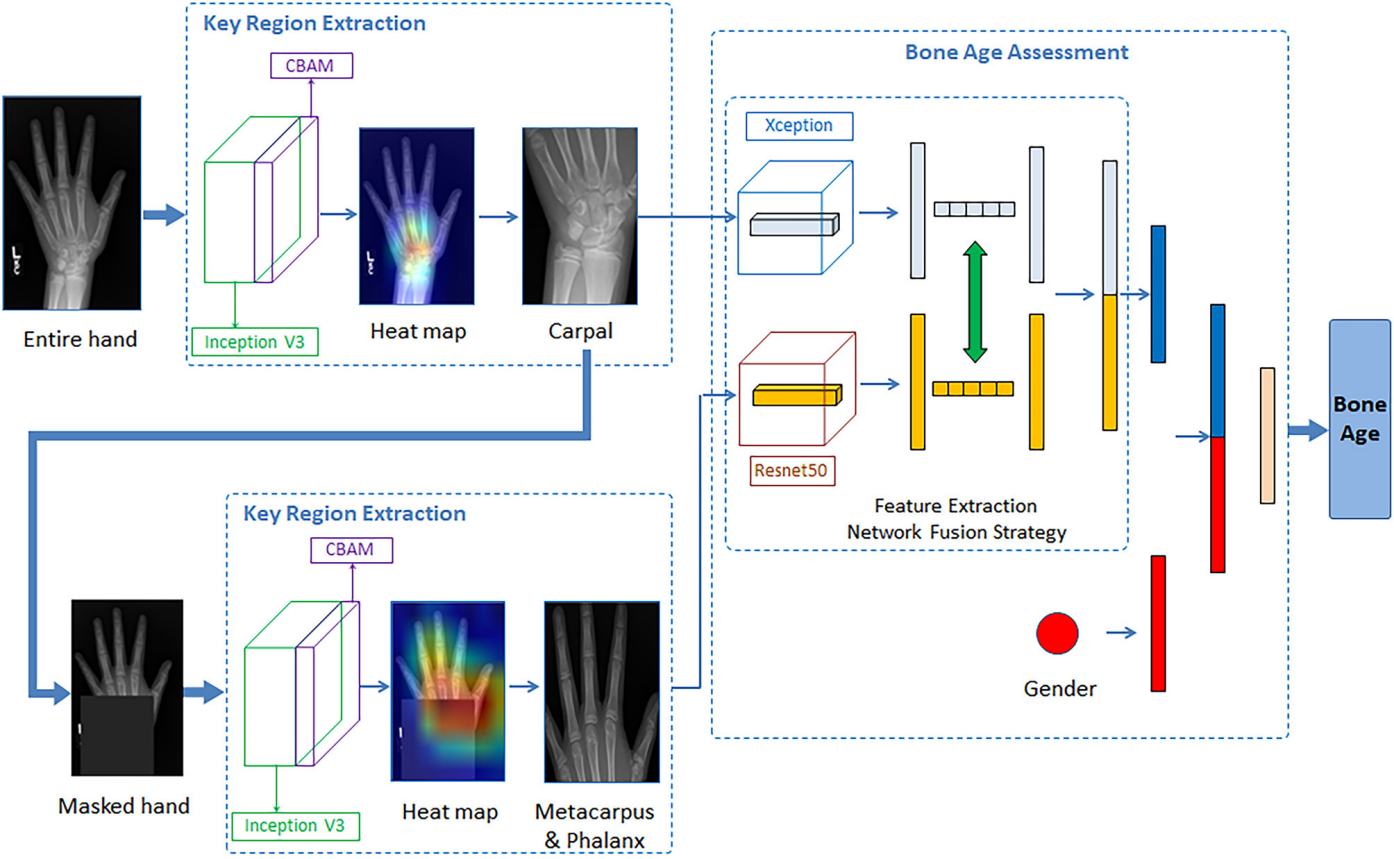
Illustration of the proposed network architecture for the BAA.

In this section, we introduce the architecture of the proposed two-stage method, which composes a critical bone region extraction network and a bone age recognition network. Specifically, the whole hand image data was fed into an improved Inception V3 network with CBAM as input, generating visual heat maps. The peak attention area of the heat map could be considered a critical bone region, and such a critical bone region (carpal area) was cropped and preserved. Then the original image was masked by a black rectangle on the critical bone area. The masked image went through the critical region extraction network again, and another critical bone region (metacarpal and finger area) could be extracted. For the bone age recognition network, automatically cropped images are input into the Xception model and ResNet50 model for feature learning. Then, according to the mid-fusion method, the carpal bone features extracted from the Xception model and the metacarpus&phalanx bone features extracted from the Res-Net50 model are exchanged and concatenated. The gender information is then entered into the second network as additional input. Finally, the concatenate feature vectors were sent into the fully connected layer for bone age estimation. The BAA network architecture is shown in Figure 2.

### Critical bone region extraction network

**Figure 3:**
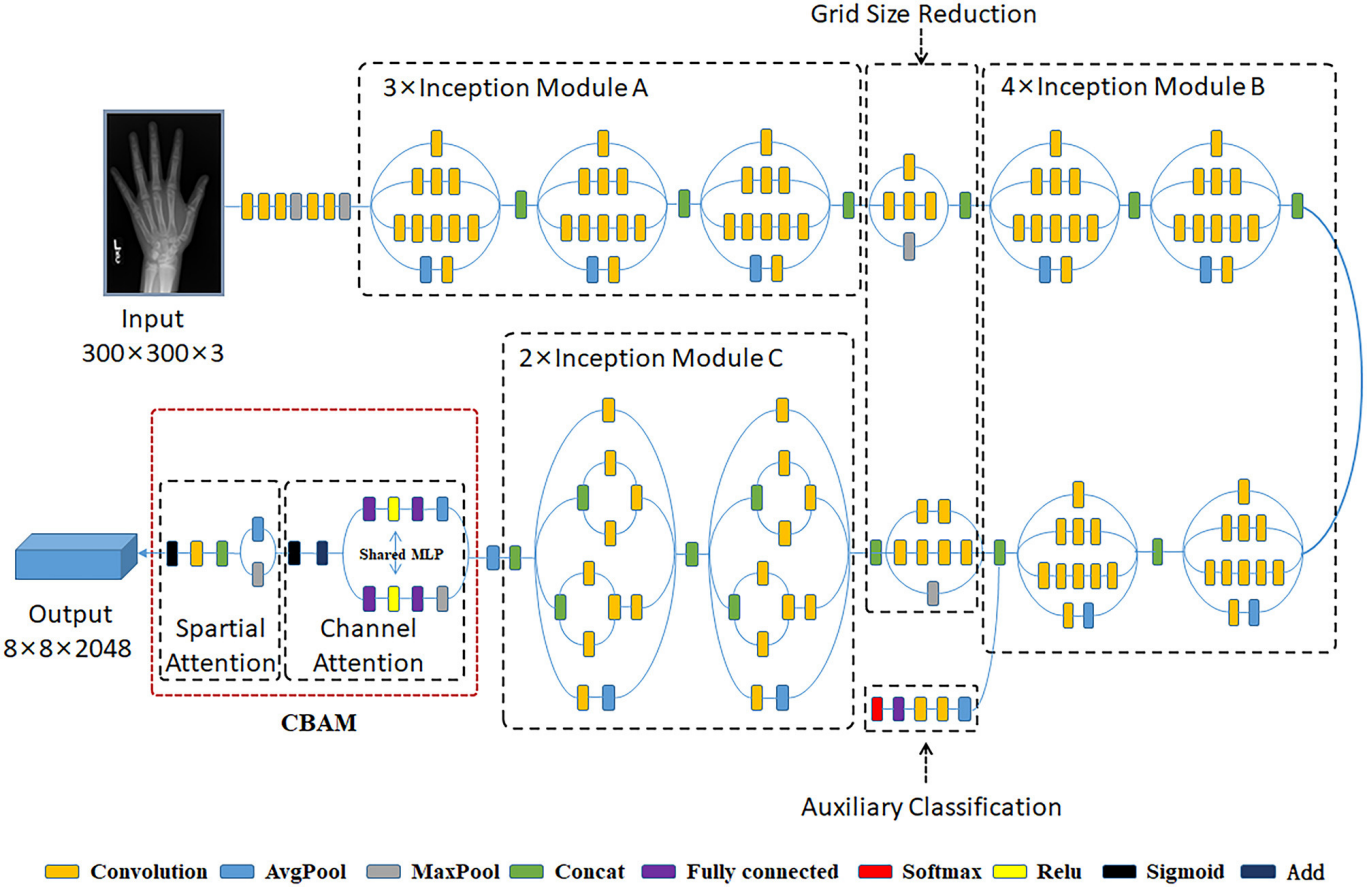
The architecture of Inception V3 with Convolutional Block Attention Module.

To better extract the important bone regions of the hand bone images and achieve an accurate assessment, we embedded the Convolutional Block Attention Module (CBAM) attention mechanism module into the Inception V3 architecture. This specific model can fully automatically detect and extract the critical bone regions *via* the peak attention area of the generated visual heat map. Figure 3 demonstrates the architecture of Inception V3 with the Convolutional Block Attention Module.

The Inception V3 architecture, proposed by Szegedy, is an advanced pre-trained CNN model (Szegedy et al., 2016). This model comprises 316 layers and 350 connections. There are 94 convolution layers of different filter sizes, where the size of the first input layer is 300 × 300 × 3. The Inception V3 is a network with good local topology because it consists of symmetrical and asymmetrical building blocks, where each block comprises multiple convolution operations, average pooling, max pooling, concatenations, and fully connected operations. In Xception, convolutional operations are employed to compress the original data, and various types of filters also are utilized on each depth space. Besides, this model benefits from the auxiliary classifier module within the medial layers to improve the discrimination capability in the lower layers. And batch normalization is commonly used and applied to the activation layer input into this model.

**Figure 4:**
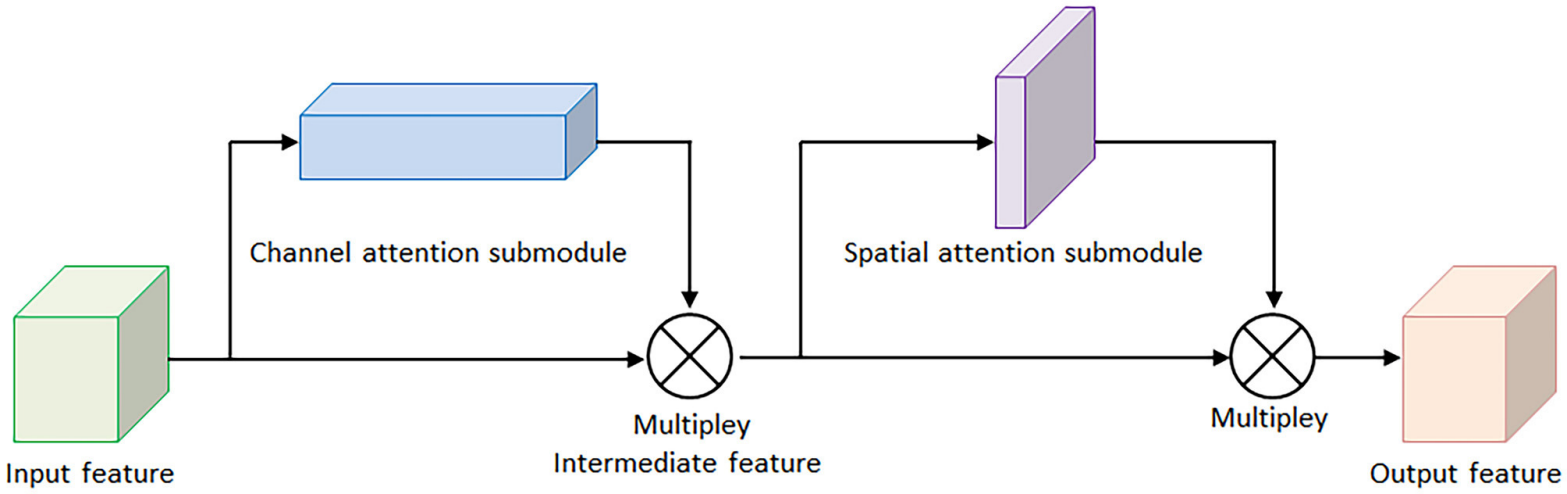
The architecture of convolutional block attention module.

For the sake of saving computing power and extracting the most useful features quickly, the attention mechanism shows an effective performance in promoting the feature-extracting capability, and it concentrates restricted attention on the important features and neglects other unnecessary information automatically. Specifically, CBAM (Woo et al., 2018) is an attention mechanism module that combines two independent submodules, including a Channel Attention Module (CAM) and a Spatial Attention Module (SAM). These two submodules are capable of improving the segmentation performance and ensuring the neural network focuses on the small target, respectively. After achieving the input feature data from the Inception V3 model, the feature data is sent to the CBAM module. Firstly, the feature data is fed to the channel attention submodule for calculation, leading to the channel attention feature data. Secondly, the channel attention feature data is multiplied by input feature data to generate the refined feature data. Thirdly, the refined feature data is sent to the spatial attention submodule and generated the spatial attention feature data. Finally, the output feature data is obtained by multiplying the spatial attention feature data with the refined feature data. The architecture of the CBAM module is shown in Figure 4.

**Figure 5:**
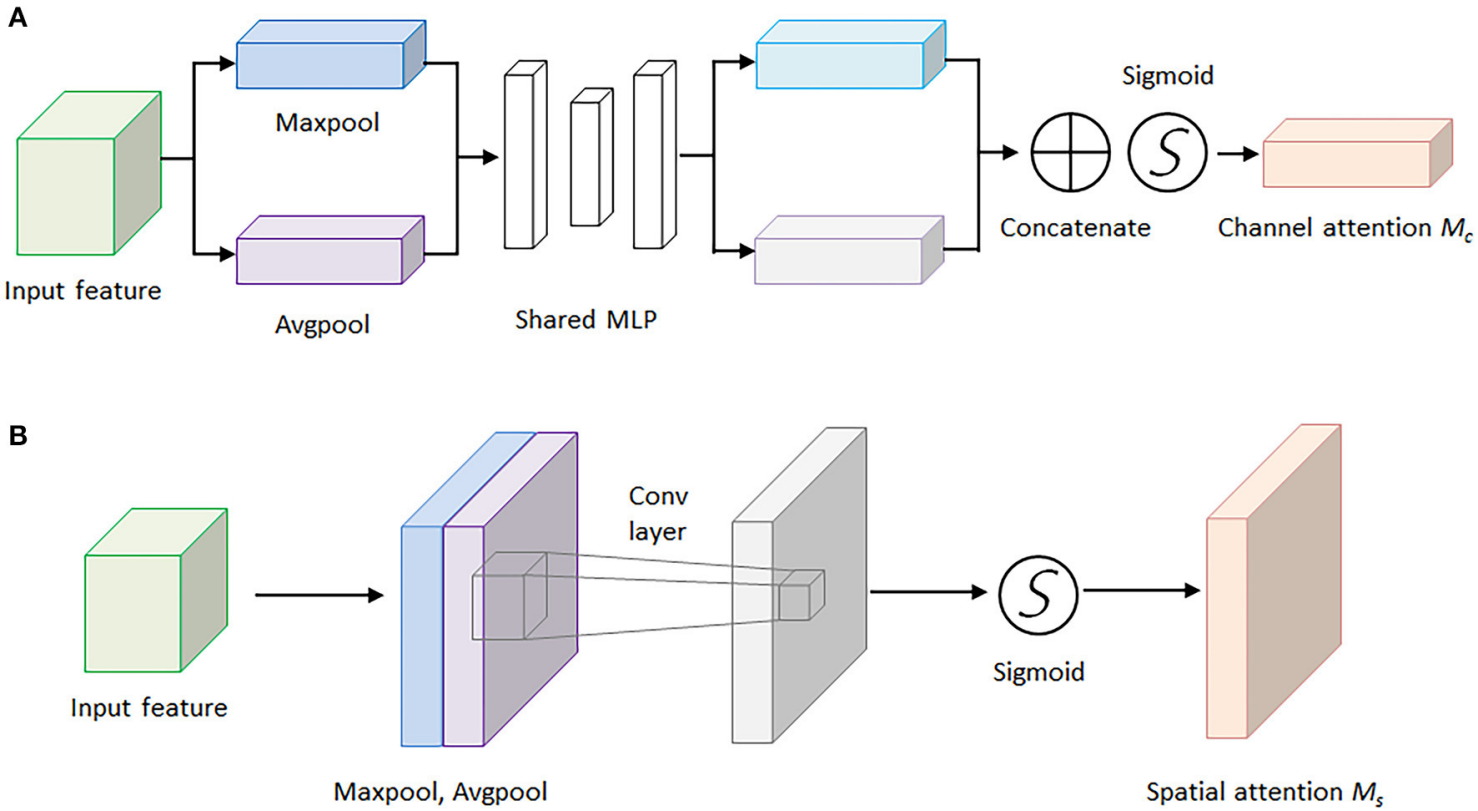
The architectures of the channel attention module and spatial attention module. **(A)** Channel attention module. **(B)** Spatial attention module.

Here, Figure 5A demonstrates the computing process of CAM, and each CAM input feature data is followed by a global maximum pooling and a global average pooling simultaneously, and the two pooling layers undergo a shared multilayer perceptron (MLP). Then, the two obtained intermediate feature layers are followed by a sum operation and a sigmoid activation operation.

On the other hand, Figure 5B illustrates the computing process of SAM, and the output feature data from the CAM is sent to the SAM submodule. A global maximum pooling and a global average pooling are conducted in order. Then the obtained intermediate data is followed by a 7 × 7 convolutional operation and a sigmoid activation operation. The channel attention weights Mc(F) and spatial attention weights Ms(F) are represented as follows:


(1) $$\begin{array}{l} M_{c}\left(F\right)=\;\sigma \left(MLP\left(AvgPool\left(F\right)\right)+MLP\left(MaxPool\left(F\right)\right)\right)\\ \;=\;\sigma \left(W_{1}\left(W_{0}\left(F_{avg}^{c}\right)\right)+W_{1}\left(W_{0}\left(F_{\max}^{c}\right)\right)\right) \end{array}$$

(2) $$\begin{array}{l} M_{s}\left(F\right)=\;\sigma \left(f^{7\times 7}\left(\left[AvgPool\left(F\right);MaxPool\left(F\right)\right]\right)\right)\\ \;=\;\sigma \left(f^{7\times 7}\left(\left[F_{avg}^{s};F_{\max}^{s}\right]\right)\right) \end{array}$$

where F represents the output feature map for each layer of the model, MLP is the fully connected layer, AvG&Pool is the global average pooling layer, MaxPool is the global maximum pooling layer, and σ is the sigmoid activation function. The spatial attention weights are calculated in a similar way, but the pooling layer is changed to be a channel domain pooling, and the MLP layer is changed to be a convolution layer, where **f** (7 × 7) represents 7 × 7 convolution layer.

In the critical bone region network, we omit the top layer of the Inception V3 network, then the remaining architecture is adopted as the backbone network for feature extraction, and the model is loaded with pre-trained weights on the ImageNet dataset. Applying a modified CAM method, the grad-CAM method, visualized heat map of the hand bone image is generated. In light of the peak attention of visualized heat maps, the important bone region (including the carpal bone region and the metacarpal finger bone region) could be localized.

### Bone age assessment from hand bone images

**Figure 6:**
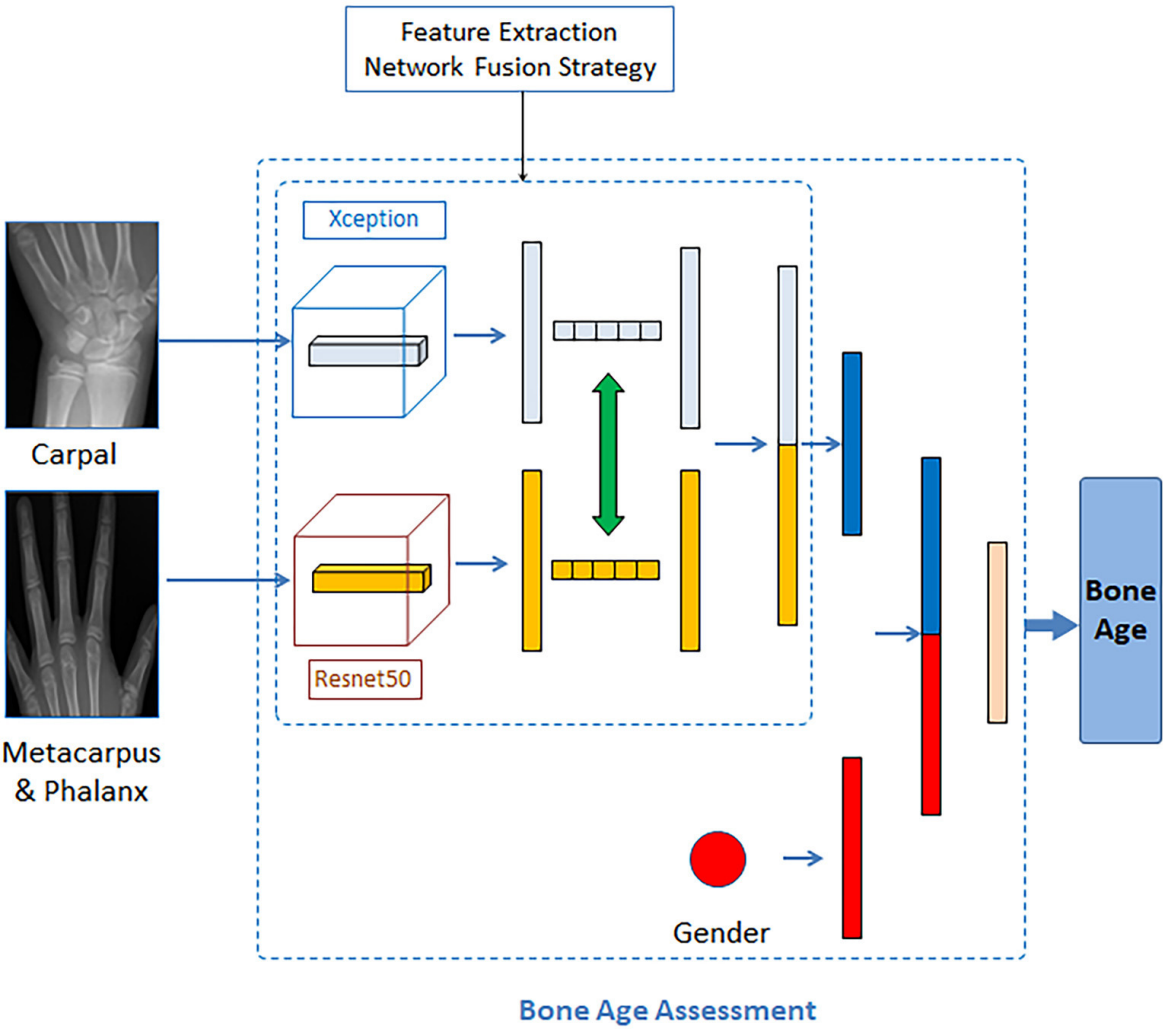
Bone age assessment network.

In this assessment module, the backbone network for feature extracting was built using the mid-term fusion method, which refers to the first transformation of different modal data into high-dimensional feature expression, and then fusion in the middle layer of the model (Wang et al., 2020). Combined with the basic principles of the mid-term fusion method, we used the Xception network and ResNet50 network to extract the bone region features of the carpal and metacarpus&phalanx regions, respectively. Then we extract the modal fusion information through the dynamic exchange of feature mappings. The method of exchanging feature information is to exchange the car-pal bone information extracted by Xception and the metacarpus&phalanx bone information extracted by ResNet50 as a whole and then concatenate the exchanged feature information. Finally, the obtained characteristics of key bone regions and gender information were concatenated and input into the last fully connected layer to output the age of hand bones. The bone age assessment network is shown in Figure 6.

**Figure 7:**
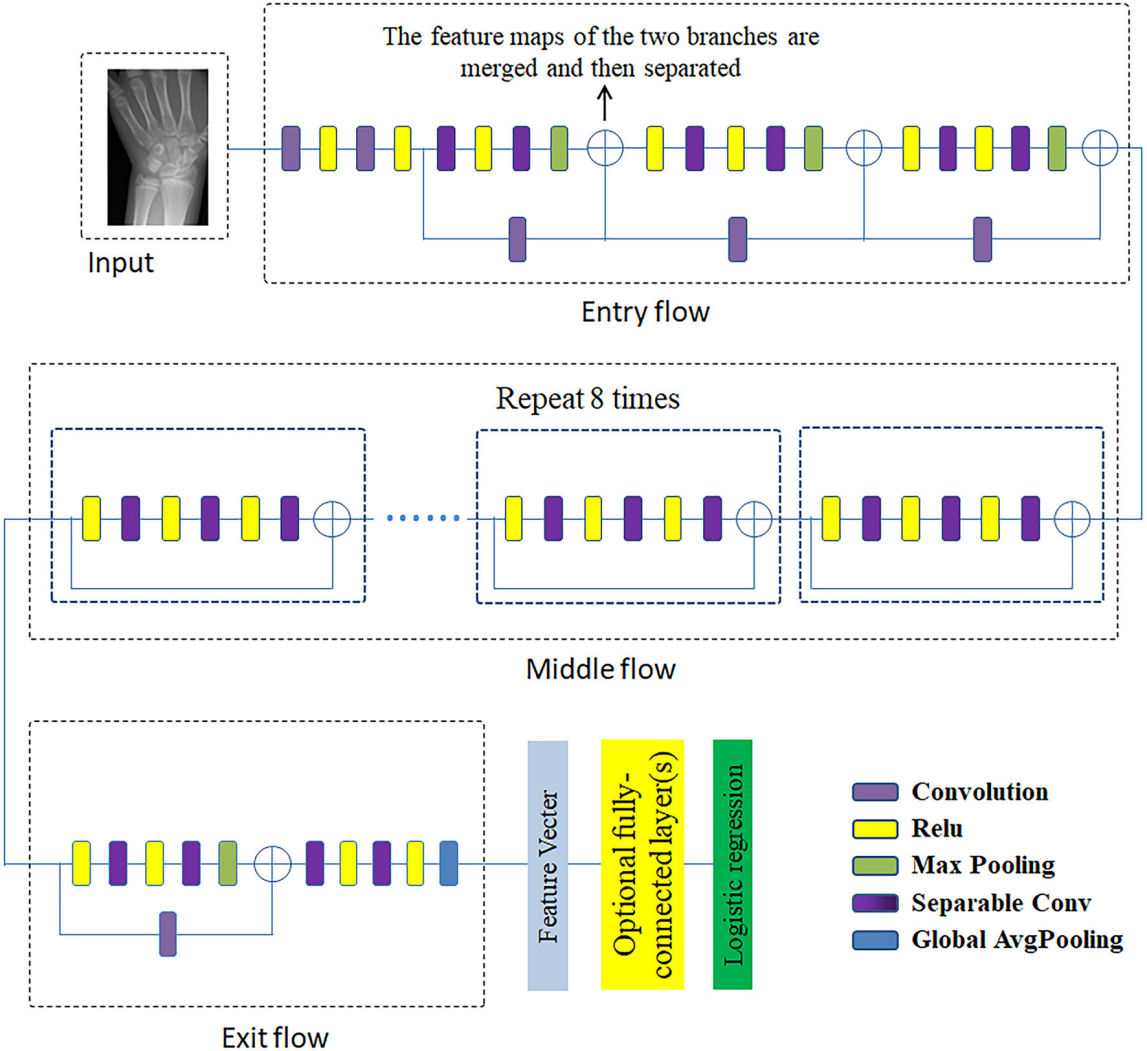
The architecture of Xception network.

The Xception network (Chollet, 2017) has been modified to remove the top layer and is followed by a convolution layer and a maximum pooling layer. The architecture of the Xception network is shown in Figure 7. The Xception architecture is composed of an entry flow module, a middle flow module (repeat 8 times), and an exit flow module. Compared to the Inception network, the Xception network first uses filters on each depth map and then condenses the data space *via* convolution operation across the depth. Therefore, this network is capable of revealing the spatial relationships for each output channel separately and extracting the cross-channel relationship. The Xception model is a linear amount of depthwise separable convolution layers with residual connections, and the depthwise separable convolution is another version of traditional convolutions that contributes to decreasing the computational time. There are 36 convolution layers in this architecture for feature learning and they constitute 14 modules. Excepting the first and last modules, the remaining modules have linear residual connections. In addition, both the convolution layer and depthwise separable convolution layers are followed by a ReLU layer.

**Figure 8:**
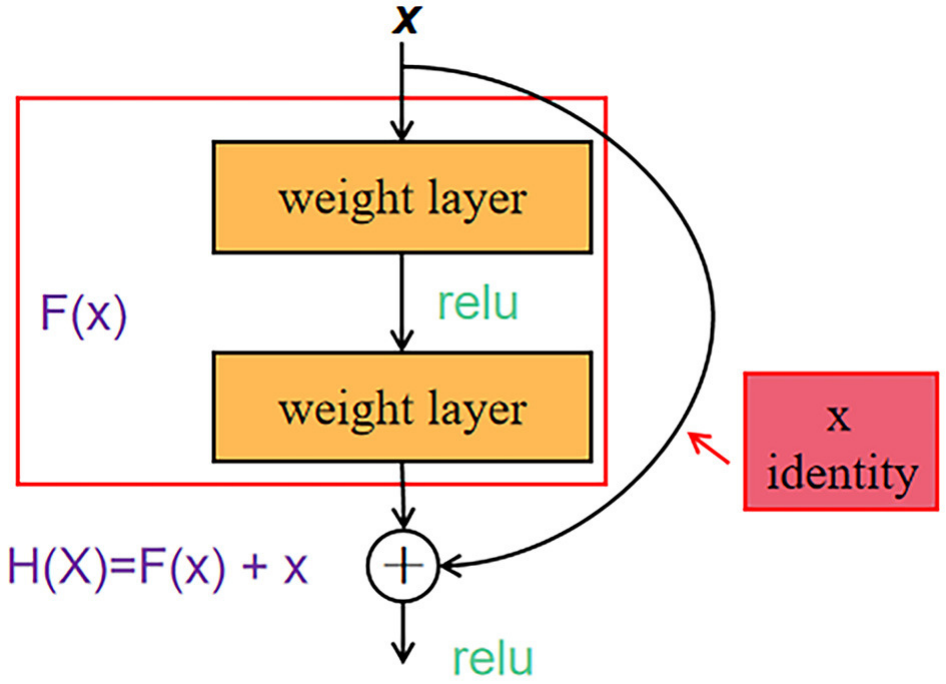
ResNet50 residual learning unit structure.

ResNet50 is a representative network of ResNet residual network series through the short-circuit mechanism (He et al., 2016). The structure of the residual learning unit is shown in Figure 8. Through the introduction of identity mapping, the residual learning unit establishes a direct correlation channel between input and output, which makes the powerful reference layer concentrate on learning the residual between input and output. An important design principle of ResNet is that when the feature map size is reduced by half, the number of feature maps is doubled, which maintains the complexity of the network layer.

Because of the obvious discrepancies between male and female radiographs with the same bone age assessment, gender provides critical information for bone age assessment. Therefore, the auxiliary network inputs the gender information into the encoder to obtain the corresponding gender features. After concatenating feature maps extracted from the backbone network $x_{D}^{l}$ and feature maps extracted from the auxiliary network $x_{G}^{l}$ in an *l*th layer, we utilize the backbone network to train the combined data and send the feature maps to the last fully connected layer of the backbone network.


(3) $${\tilde{x}}_{D}=\propto_{D}^{l}x_{D}^{l}+\propto_{G}^{l}x_{G}^{l}\;$$

Where ${\tilde{x}}_{D}$ denotes the assessment bone age result of the corresponding subject, the $\propto_{D}^{l}$ and $\propto_{G}^{l}$ are learnable weights in the *l*th layer of the assessment network and $\propto_{G}^{l}$ reveals the contribution of gender information.

### Loss function and evaluation metrics

In this experiment, the neural network was trained on the RSNA dataset and the additional dataset, respectively. The Inception V3 model loaded with pre-trained weights on the ImageNet dataset is employed as the backbone and the prediction network was used by the Xception model and Resnet50 model. A linear transformation was employed for the gender information, and it was considered as an additional input of the prediction network. In the training process, the input hand image is preprocessed, namely, resampling, rotating, and changing contrast, and brightness, to prevent overfitting. The size of the initial image is set to (300, 300, 3), the size of the learning rate is set to 0.0003, and the number of epochs for training is set to 30.

Due to the proposed model regards the bone age assessment as a regression task, mean square error (MSE) is adopted as the loss function in this task,


(4) $$L_{MSE}=\frac{1}{n}\sum_{i=1}^{n}{\left|y_{i}-g_{i}\right|}^{2}$$

where n represents the number of the training sets, *y*_*i*_ is the ground-truth age and *g*_*i*_ is the predicted value of bone age.

In this study, bone age assessment was treated as a regression task, the evaluation indicator for network performance is the mean absolute error (MAE) between the output of the model and the ground-truth age, which is represented as


(5) $$MAE=\frac{1}{N}\sum_{i=1}^{N}\left|y_{i}-\hat{y_{i}}\right|$$

where N represents the number of input samples and *y*_*i*_ is the true value of bone age, and $\hat{y_{i}}$ is the predicted bone age.

## Results and discussion

**Figure 9:**
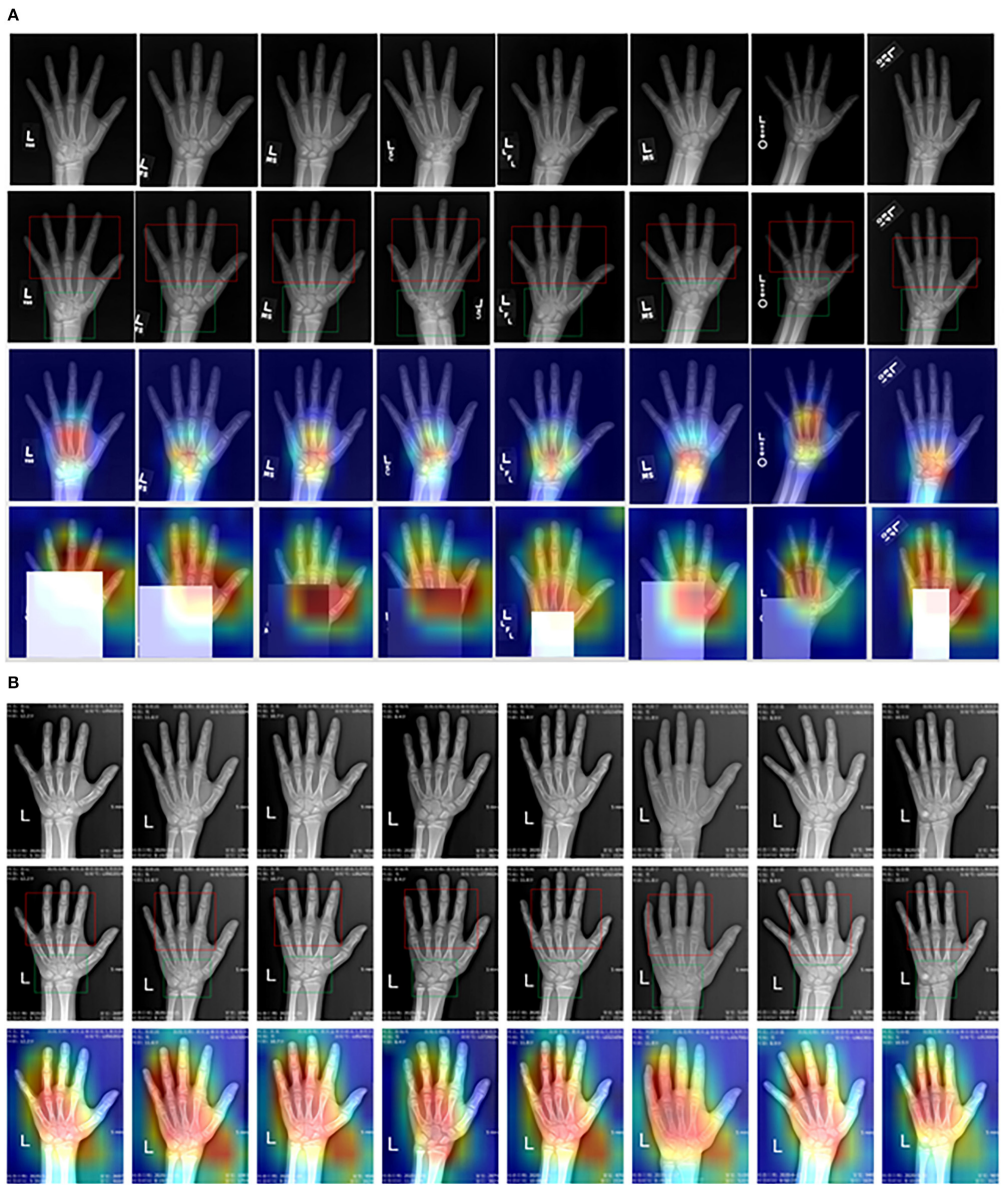
Visual heat map of critical bone region **(A)** RSNA dataset, **(B)** CQJTJ dataset.

In this study, we present a deep neural network incorporating CBAM to address the limitations of neglecting important regions in hand bone images and the costly manual annotation process. The results of the ROI extraction and feature activation heat map are presented in Figure 9. To evaluate the performance of the part extraction module, visual maps were generated using the Inception V3 model with CBAM. The extracted hand regions were then outlined with red and green bounding boxes on the maps, with the green boxes indicating the carpal bones and the red boxes indicating the metacarpal and phalanx bones. This supports conventional bone age assessment theories by clearly distinguishing between these regions. The proposed method extracts key bone areas in an explainable and reasonable manner, with the part extraction module effectively removing background distractions, thereby improving the robustness of the method without additional segmentation.

Meanwhile, we evaluated the visual interpretation of the feature-extracting network through the grad-CAM method and examined the activated heat maps on the original images. The third and fourth rows in Figure 9A show activated heat maps on the RSNA dataset. The red-colored areas indicate high attention and the results reveal that the network has broad activation over the carpal and metacarpal areas with a peak focus on the carpal area initially. Masking the carpal part of the input images results in an expanded activation region to the entire hand, and the peak attention shifts to the metacarpal finger area. On the other hand, Figure 9B displays activated heat maps on our private CQJTJ dataset, which reveals activation across almost the entire hand with peak attention on the critical hand bone areas including carpals, metacarpals, and fingers. This difference in activation could be attributed to the improved image quality and resolution in our private CQJTJ dataset. Essentially, the visualized heat maps confirm that the proposed neural network is attending to the critical locations, aligning with conventional clinical approaches that focus on carpals, metacarpals, and phalanx. In comparison, the ResNet50 method, a ROI-free approach, concentrate activation on the metacarpal areas, which may limit recognition learning and make it tendentious (Liu et al., 2020). Thus, the feature activation from the proposed deep network is explainable. In conclusion, the visual explanation suggests that the proposed part-extracting network achieves better and more effective feature learning performance about important bone areas, leading to improved bone age estimation.

**Figure 10:**
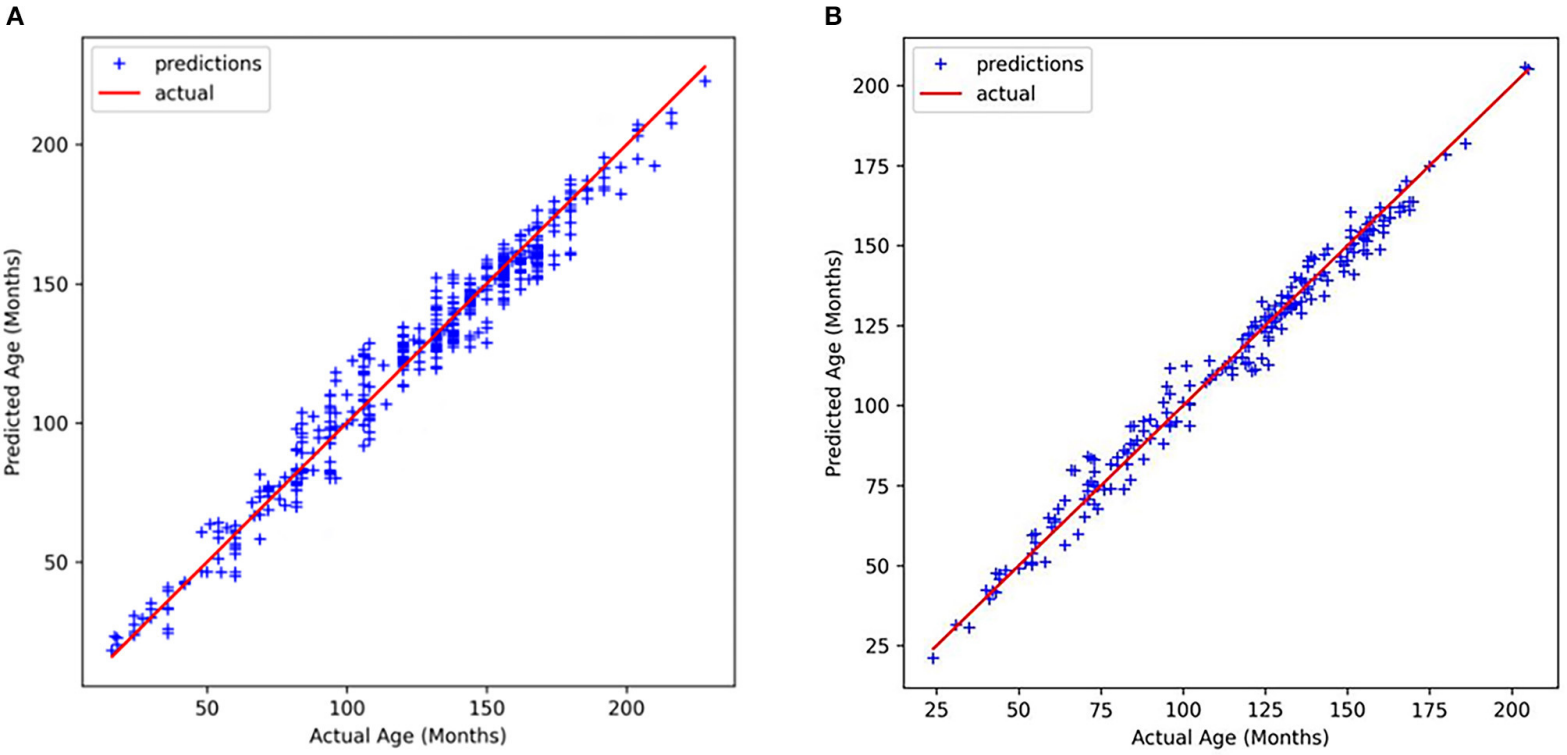
Relationship between actual age and predicted age **(A)** RSNA dataset, **(B)** CQJTJ dataset.

After obtaining the critical bone areas, we fed the critical bone images into the backbone network for feature learning and age prediction. The correlations between the true age and the estimated age are shown in Figure 10. It is obvious that our predicted ages are strongly consistent with the true age. Specifically, Figure 10A illustrates the correlation results on the public data set RSNA, and Figure 10B illustrates the correlation results on the private CQJTJ data set. In our research, we utilize MAE to estimate the performance of our proposed approach. The assessment performance results show the MAE of 5.45 months on the public RSNA dataset, and the MAE of 3.34 months on the private CQJTJ dataset. As analyzed above, the improved performance on the private CQJTJ dataset can be attributed to the higher quality and resolution of the images, as well as the homogeneity of the subjects, which consist of a single race. This is consistent with the known fact that bone maturation varies based on race.

We also compare the assessment performance of our proposed method with other existing advanced networks, as illustrated in Table 1. Particularly, the ROI-free methods reported by Larson et al. (2017), Spampinato et al. (2017), Pan et al. (2020), Li et al. (2022), respectively, employ the entire hand images as input without any additional manual annotations. The ROI-based approaches reported by Iglovikov et al. (2017), Ren et al. (2018), Zhao et al. (2018), Son et al. (2019), respectively, utilize critical bone area images as input with precise ROI annotations. The method proposed by Iglovikov et al. (2017) requires the use of additional CNN to perform pre-processing steps, as well as a manual annotation to obtain hand bone mask images of hand bone images. Each mask image takes 2 min. At the same time, in the training process of removing the background and improving the accuracy of segmentation results, it is also necessary to manually label the images with poor hand bone quality 6 times. Compared with the method proposed by Iglovikov et al. (2017) the method proposed by Ren et al. (2018) regards bone age assessment as a regression task. The method they proposed only takes 1.5 s to complete one bone age assessment, but when removing the influence of background irrelevant factors, 1,000 rectangular boundary boxes need to be manually marked to remove the irrelevant background. Iglovikov and Ren's method has good accuracy, but they require manual annotation of bone images, and this additional manual annotation is time-consuming and expensive. To overcome the time-consuming and expensive problem of additional manual annotation, CBAM, a plug-and-play attention mechanism, can be added to better automatically locate the key bone region of the hand bone image. Meanwhile, Grad-CAM is used to generate visual heat maps of the carpal bone and metacarpus&phalanx, which is also conducive to an intuitive comparison between physicians and traditional mapping methods. It further demonstrated the excellent clinical acceptance of this study method. Generally speaking, ROI-based methods demonstrate better performance in BAA than ROI-free methods, as shown in Table 1. As mentioned above, the additional manual annotation is tedious and time-consuming. Furthermore, Wu et al. (2018), Mehta et al. (2021), Yang et al. (2021) and our method detects the specific bone area automatically without any additional manual annotations and fed the critical bone area images into the age prediction network as input. In general, the experimental results show that our method performs better than the majority of current evaluation methods without additional labor annotation costs.

**Table 1:** The results of comparison with other methods on RSNA dataset.

**Method**	**Dataset**	**No critical bone-areas**	**Additional annotations**	**MAE (months)**
Spampinato et al. (2017)	RSNA	√	×	9.12
Larson et al. (2017)	RSNA	√	×	6.24
Marouf et al. (2020)	RSNA	√	×	6
Pan et al. (2020)	RSNA	√	×	8.59
Li et al. (2022)	RSNA	√	×	6.2
Ren et al. (2018)	RSNA	×	√	5.2
Zhao et al. (2018)	RSNA	×	√	7.66
Iglovikov et al. (2017)	RSNA	×	√	4.97
Son et al. (2019)	RSNA	×	√	5.52
Wu et al. (2018)	RSNA	×	×	7.38
Yang et al. (2021)	RSNA	×	×	6.14
Mehta et al. (2021)	RSNA	×	×	5.92
Ours	RSNA	×	×	5.45

MAE, mean absolute error.

To reinforce the efficacy of the proposed method and emphasize the importance of incorporating gender information, we conducted a comparative study on the RSNA dataset. The results of the experiment, which compared the impact of attention mechanisms and gender information on prediction outcomes, are presented in Table 2.

**Table 2:** Effect of attentional mechanism and gender information on prediction results.

	**Original image**	**Carpal**	**Metacarpus and phalanx**	**Carpal + metacarpus and phalanx**
No Gender + No CBAM	8.60	7.85	11.08	7.78
Gender + No CBAM	5.95	6.20	6.71	5.71
No Gender + CBAM	—	7.67	8.66	7.30
Gender + CBAM	—	5.50	6.23	5.45

CBAM, Convolutional Block Attention Module.

The results presented in Table 2 demonstrate the significance of incorporating gender information in bone age evaluation. The accuracy of the evaluation, measured as MAE, was found to decrease from MAE 5.95 months to MAE 8.60 months when gender information was removed. This highlights the importance of considering gender as a factor in bone age assessment. Additionally, the results show that the proposed method, which utilizes the combination of the carpal region, metacarpus and phalanx region, and gender information as input, outperforms other methods, with a MAE of 5.45 months on the public RSNA dataset and 3.34 months on a private dataset. Furthermore, the results indicate that the utilization of the CBAM attention mechanism in the recognition network can improve performance, as it emphasizes relevant features and suppresses irrelevant information. The optimal result was achieved by combining both the attention mechanism and gender information, demonstrating that they are complementary and can be employed together to enhance accuracy in bone age recognition.

## Conclusion

In this study, we introduce a novel two-stage, fully automatic method for evaluating bone age without the requirement for manual annotations. Our results demonstrate that the localization of critical bone regions can significantly improve the accuracy of bone age evaluation, and gender information plays a crucial role in this process. The proposed method addresses the issue of extracting key hand bones and reduces the dependence on costly and subjective manual annotations. Consequently, it achieves an acceptable level of accuracy and interpretability and has the potential for eventual clinical implementation. We believe that this proposed method holds great promise for future clinical applications. However, the research in the field of bone age assessment can be expanded and improved from the following aspects: This study demonstrated the influence of key bone regions on the prediction results but did not further discuss the influence of key areas such as carpal bone and metacarpus&phalanx bone on the accuracy of bone age prediction. In future studies, specific regions can be extracted by positioning network for network model training to further demonstrate the influence of different bone regions in the field of bone age prediction. At the same time, the experimental effect achieved in this study is the two-stage separation mode of positioning and prediction. In the later stage, it can be considered to integrate positioning and prediction into an end-to-end network. This end-to-end network not only has strong clinical acceptance but also can be better applied in the field of bone age evaluation, helping physicians realize accurate bone age evaluation.

## Data availability statement

The datasets presented in this study can be found in online repositories. The names of the repository/repositories and accession number(s) can be found below: https://www.kaggle.com/kmader/rsna-bone-age.

## Ethics statement

Ethical review and approval was not required for the study on human participants in accordance with the local legislation and institutional requirements. Written informed consent to participate in this study was provided by the participants' legal guardian/next of kin. Written informed consent was obtained from the individual(s), and minor(s)' legal guardian/next of kin, for the publication of any potentially identifiable images or data included in this article.

## Author contributions

ZL was responsible for providing the ideas and expenditure in the project. WC mainly completed the work of locating key bone areas and constructing the network for predicting the age of hand bone. YC completed the work of data preprocessing. YJ and ZH helped WC to build the network. XL and YJ provided the modification of the thought to increase the degree of innovation in the research. All authors contributed to the article and approved the submitted version.
